# Autism and Neurodegeneration: Distinct Disorders or a Shared Biological Continuum?

**DOI:** 10.3390/brainsci16070766

**Published:** 2026-07-21

**Authors:** Jorge Manzo, María Elena Hernández-Aguilar

**Affiliations:** Instituto de Investigaciones Cerebrales, Universidad Veracruzana, Xalapa 91190, Veracruz, Mexico; elenahernandez@uv.mx

**Keywords:** autism spectrum disorder, neurodegeneration, lifespan continuum, mitochondrial dysfunction, neuroinflammation, proteostasis, excitation–inhibition balance, cerebellum

## Abstract

**Highlights:**

**What are the main findings?**

- Autism spectrum disorder and neurodegenerative diseases are distinct clinical entities that share partially overlapping biological mechanisms, supporting a model of convergence within a lifespan continuum rather than complete separation.
- Convergent evidence across genetic, molecular, circuit, and epidemiological levels indicates that early neurodevelopmental alterations may establish latent vulnerabilities that, under specific conditions, intersect with neurodegenerative processes later in life.

**What are the implications of the main findings?**

- Autism should be considered within a lifespan framework, integrating pediatric, adult, and geriatric perspectives to improve monitoring of long-term neurological health and identify at-risk subgroups.
- Targeting shared biological mechanisms may enable the development of longitudinal biomarkers and early interventions aimed at enhancing neural resilience and reducing vulnerability to neurodegenerative outcomes.

**Abstract:**

Background/Objectives: Autism spectrum disorder (ASD) is traditionally classified as a neurodevelopmental condition, whereas neurodegenerative diseases are defined by progressive neuronal decline in later life. This separation has shaped research and clinical practice, yet emerging evidence suggests potential biological overlap. This review aims to evaluate whether ASD and neurodegenerative disorders represent distinct entities or are linked through shared mechanisms operating across the lifespan. Methods: This narrative review synthesizes findings from genetic, molecular, cellular, circuit-level, and epidemiological studies examining ASD and major neurodegenerative conditions, including Alzheimer’s disease, Parkinson’s disease, and Amyotrophic lateral sclerosis. Emphasis is placed on identifying convergent pathways and evaluating evidence within a lifespan-oriented framework. Results: Across multiple levels of analysis, ASD and neurodegenerative diseases share partially overlapping biological mechanisms, including mitochondrial dysfunction, impaired proteostasis, neuroimmune alterations, and network-level instability. Genetic and molecular data reveal pleiotropic pathways influencing both early neurodevelopment and later neuronal resilience. Circuit-level studies highlight shared principles of network vulnerability, including cerebellar involvement and excitation–inhibition imbalance. Epidemiological data further indicate increased risk of dementia and parkinsonian features in autistic adults. These convergences suggest that early neurodevelopmental alterations may establish latent vulnerabilities that, under specific conditions, intersect with neurodegenerative processes later in life. Conclusions: ASD and neurodegenerative diseases are best understood as distinct clinical conditions that share partially overlapping biological substrates. Rather than implying a deterministic progression, the evidence supports a model of lifespan convergence in which timing, context, and individual susceptibility shape outcomes. This framework highlights the need for integrated research and clinical approaches that consider brain health as a continuous process from development through aging.

## 1. Introduction

Autism spectrum disorder (ASD) and neurodegenerative diseases (NDDs)—including Alzheimer’s disease (AD), Parkinson’s disease (PD), Amyotrophic lateral sclerosis (ALS), and Frontotemporal dementia (FTD)—have traditionally been conceptualized as conditions occupying opposite ends of a temporal and biological spectrum. ASD is classically defined as a neurodevelopmental condition arising during early embryonic and postnatal brain maturation, characterized by atypical neural connectivity, imbalances in excitatory–inhibitory circuitry, and alterations in synaptogenesis, pruning, and plasticity during sensitive developmental windows [1,2,3]. In contrast, NDDs are typically understood as disorders of later life, marked by the progressive accumulation of pathogenic protein aggregates (Aβ and tau in AD; α-synuclein in PD; TDP-43 in ALS and FTD), sustained neuroinflammation, mitochondrial decline, and ultimately neuronal loss that emerges in mid-to-late adulthood [4,5,6,7]. This dichotomous framework—neurodevelopmental disruption versus neurodegenerative deterioration—has long shaped diagnostic categories, clinical expectations, and therapeutic strategies.

However, this traditional separation is increasingly being reconsidered. A growing body of evidence indicates that several biological features commonly associated with neurodegeneration are also detectable, albeit in different contexts and temporal patterns, in ASD. Neuroanatomical and biochemical studies have reported alterations such as regionally selective neuronal loss—most notably reductions in cerebellar Purkinje cells—along with persistent microglial activation, elevated pro-inflammatory cytokines in brain tissue and cerebrospinal fluid, and biochemical markers of oxidative and nucleic acid damage, including increased 3-nitrotyrosine and 8-oxo-deoxyguanosine [8]. While these findings do not imply that ASD is a neurodegenerative disorder, they challenge the notion that the underlying biology of these conditions is entirely distinct.

These observations invite a reframing of the relationship between ASD and NDDs. Rather than representing mutually exclusive categories, they may reflect different temporal expressions of partially overlapping biological processes that unfold across the lifespan. From this perspective, early neurodevelopmental alterations—affecting synaptic organization, metabolic regulation, immune signaling, and cellular stress responses—could shape the long-term resilience or vulnerability of neural systems. Over time, and under specific genetic or environmental conditions, these early perturbations may intersect with mechanisms classically associated with neurodegeneration, such as impaired proteostasis, chronic inflammation, and mitochondrial dysfunction.

Importantly, this conceptual shift does not suggest a uniform or inevitable progression from ASD to neurodegeneration. Instead, it raises a more nuanced question: whether certain biological trajectories within the heterogeneous spectrum of ASD may predispose to, or converge with, neurodegenerative processes later in life. In this sense, the focus moves from categorical distinctions to lifespan dynamics, where vulnerability, compensation, and adaptation interact over time.

The sections that follow develop this framework by examining evidence for convergence across multiple levels of analysis, including genetic architecture, molecular pathways, neural circuitry, and epidemiological patterns. Through this integrative approach, the present work seeks to reassess whether ASD and major NDDs are best understood as distinct disorders, or as conditions linked—at least in part—by a shared and evolving biological continuum.

## 2. Genetic Architectures Linking Autism and Neurodegeneration

The genetic boundary traditionally separating ASD from NDDs is becoming increasingly permeable as shared biological functions are uncovered. Rather than reflecting entirely distinct etiologies, a growing number of genes implicated in ASD also participate in cellular processes that are central to neuronal maintenance and survival across the lifespan. This overlap suggests that genetic risk may not be confined to a single developmental window but instead shape trajectories of vulnerability that extend from early brain formation into aging. A representative example is *ADNP* (activity-dependent neuroprotective protein), a gene essential for embryonic brain development and cognitive function, whose pathogenic variants are strongly associated with ASD and intellectual disability. Beyond its developmental role, *ADNP* regulates autophagy, a process critical for neuronal proteostasis and long-term cellular integrity. Disruption of this pathway introduces a persistent vulnerability, as impaired autophagic clearance can compromise neuronal resilience over time. Consistent with this dual role, *ADNP* dysfunction has been linked not only to ASD but also to schizophrenia, Alzheimer’s disease, and tau-related disorders. Overall, *ADNP* dysregulation is disease-dependent. ASD and schizophrenia are generally associated with increased *ADNP* gene expression, whereas Parkinson’s disease and circulating *ADNP* protein levels in Alzheimer’s disease show reduced expression, suggesting that disruption of *ADNP* homeostasis rather than a uniform direction of expression change underlies these disorders [9].

Mitochondrial pathways provide another compelling point of convergence between neurodevelopmental and neurodegenerative biology. Recent genomic studies have identified rare exonic deletions in genes classically associated with Parkinson’s disease—including *PRKN*, *SNCAIP*, and *RABEPK*—in children with ASD [10]. These genes are involved in mitochondrial quality control, synaptic regulation, and intracellular trafficking, processes that are essential both for early neural development and for the prevention of cellular degeneration. Notably, affected individuals do not exhibit Parkinsonian features in childhood, raising the possibility that such genetic alterations represent latent vulnerabilities whose phenotypic expression may depend on time and context [10]. Further support for this perspective comes from studies of neurodevelopmental regression (NDR) in ASD, a phenomenon characterized by the loss of previously acquired skills. NDR is more prevalent in autistic children with co-occurring mitochondrial dysfunction, who display altered bioenergetic profiles, including elevated basal respiration and increased susceptibility to oxidative stress. This pattern suggests a maladaptive mitochondrial response, potentially triggered by environmental factors, that compromises neuronal stability. In this context, mitochondrial dysfunction may not only contribute to early developmental disruption but also initiate processes that resemble aspects of neurodegenerative cascades, particularly those related to energy failure and oxidative damage [11].

A broader conceptual framework linking these observations is pleiotropy, whereby a single gene influences multiple phenotypic outcomes across different stages of life [12]. Chromatin remodelers offer a clear illustration of this principle. Among them, *CHD8* has emerged as a key regulator of neurodevelopment, whose haploinsufficiency disrupts transcriptional programs with relevance to both ASD and neurodegeneration [12,13,14]. In human neural progenitors, reduced *CHD8* expression alters the regulation of more than 1700 genes, including those involved in neuronal differentiation, synaptogenesis, axon guidance, and cell adhesion. Complementary studies in murine models reveal widespread dysregulation of gene networks governing neurogenesis, RNA processing, chromatin remodeling, and neuroimmune signaling, accompanied by phenotypes such as increased neuronal proliferation, megalencephaly, and cognitive impairment. Although these models do not directly address neurodegenerative disease, the affected pathways point to enduring cellular vulnerabilities, particularly those involving chromatin regulation, neuronal maturation, and long-term synaptic stability [13,14].

Taken together, these findings suggest that ASD and neurodegenerative diseases may arise not from entirely separate genetic architectures, but from partially overlapping molecular susceptibilities that unfold across the lifespan. The marked clinical differences between early-onset neurodevelopmental conditions and late-onset neurodegeneration may therefore depend less on which genes are involved, and more on the timing, magnitude, and context of their disruption. Alterations in processes such as autophagy, for example, can have distinct consequences depending on when they occur: during early development, they may interfere with neuronal maturation and circuit formation, contributing to ASD; later in life, or in combination with age-related decline in cellular clearance mechanisms, similar disruptions may favor protein accumulation and neuronal degeneration [15].

From this perspective, autism and neurodegenerative disorders can be understood as different expressions of shared biological fragilities, revealed at distinct stages along the continuum of brain development and aging. This view does not collapse the distinction between these conditions but rather reframes it—suggesting that genetic risk operates not in isolation, but within a dynamic temporal landscape that shapes how vulnerability is expressed over time.

## 3. Molecular and Cellular Mechanisms of Convergence

At the molecular and cellular levels, ASD and NDDs engage many of the same core mechanisms that sustain neuronal function across the lifespan. Rather than relying on entirely distinct biological programs, both conditions perturb a relatively limited set of processes that enable synapses to form, adapt, and remain functional over time. These include activity-dependent protein synthesis, systems responsible for the clearance of damaged or misfolded proteins, cellular energy metabolism, and the regulation of immune responses within the brain. From this perspective, the distinction between neurodevelopmental and neurodegenerative disorders becomes less absolute, raising the possibility that differences arise not from the nature of the mechanisms involved, but from the timing and context in which they are disrupted.

A representative example is the FMRP–APP–mGluR5 pathway, which operates as a molecular interface linking synaptic activity to the production and processing of amyloid precursor protein (APP). During early development, increased activity of this pathway—as observed in ASD and Fragile X syndrome—can drive excessive synaptic growth and an imbalance in excitatory signaling. In contrast, alterations in the same molecular system later in life are associated with abnormal APP processing, accumulation of toxic protein species, and synaptic dysfunction characteristic of Alzheimer’s disease [16,17,18]. This dual role illustrates how a shared molecular network can contribute to distinct phenotypic outcomes depending on developmental stage. Importantly, this pathway intersects with broader growth-regulatory systems, including PTEN and the PI3K/AKT/mTOR cascade [19,20], as well as with autophagy, the cell’s principal mechanism for recycling damaged components. When autophagy is compromised, the consequences may diverge across the lifespan: during development, synapses may fail to mature or stabilize appropriately; during aging, similar deficits may favor the accumulation of damaged proteins and progressive synaptic loss [21].

A second level of convergence emerges from the regulation of cellular bioenergetics and oxidative balance. A substantial subset of individuals with ASD exhibits systemic mitochondrial dysfunction [11,22], suggesting that altered energy metabolism is not a secondary feature but a core component of the condition in specific subgroups. During critical periods of brain development, a chronic state of reduced energetic efficiency may impose constraints on processes such as dendritic growth, synaptic pruning, and circuit refinement. These early alterations are unlikely to be transient; rather, they may establish a persistent metabolic vulnerability that influences neuronal resilience throughout life. Comparable disturbances in mitochondrial function are well-established in neurodegenerative disorders such as PD, AD, and ALS, where defects in mitochondrial quality control—particularly impaired mitophagy—are central to disease progression [23,24]. Mitophagy is essential for maintaining neuronal homeostasis, as it enables the selective removal of damaged mitochondria in cells with high energetic demands and limited regenerative capacity. Increasing evidence indicates that similar alterations in mitochondrial dynamics and quality control are also present in ASD. In this context, insufficient clearance of dysfunctional mitochondria leads to the accumulation of reactive oxygen species, disruption of calcium homeostasis, bioenergetic inefficiency, and impaired synaptic function [25]. Studies have identified abnormalities in key mitophagy-regulating pathways, including the PINK1–Parkin axis and receptor-mediated mechanisms, resulting in the persistence of fragmented, oxidative stress-producing mitochondria that interfere with neuronal development and circuit formation [26].

Across both ASD and neurodegenerative conditions, defective mitochondrial quality control promotes a state of chronic oxidative stress, sustained neuroinflammatory signaling, and metabolic insufficiency. These shared features point to a common axis of vulnerability rooted in disrupted mitochondrial homeostasis, rather than entirely distinct disease-specific mechanisms. Within a lifespan framework, such alterations may have stage-dependent consequences: early impairment of mitochondrial function may alter developmental trajectories, whereas the same vulnerability, compounded by aging-related decline in cellular maintenance systems, may increase susceptibility to neurodegenerative processes.

Neuroinflammation represents another important point of convergence between ASD and neurodegenerative disorders. Both ASD and disorders such as Alzheimer’s disease, dementia-related neuropsychiatric syndromes, and schizophrenia exhibit chronic microglial activation accompanied by increased levels of pro-inflammatory cytokines, including IL-1β, IL-6, TNF-α, IL-8, IFN-γ, and TGF-β, as well as oxidative stress and white matter abnormalities. These shared neuroimmune mechanisms may disrupt neuronal function, synaptic homeostasis, and cognitive performance. However, although neuroinflammatory alterations are consistently reported in ASD, current evidence supports considerable biological heterogeneity, suggesting that neuroimmune dysregulation characterizes specific ASD subgroups rather than representing a universal feature of the disorder [27].

Taken together, these observations support the view that ASD and NDDs engage overlapping molecular and cellular pathways that operate continuously from development through aging. The key difference may lie not in the mechanisms themselves, but in how long they persist, how they interact with additional stressors, and when their cumulative effects exceed the brain’s capacity for compensation. In this sense, molecular convergence does not imply equivalence between conditions, but rather reveals a shared biological substrate through which distinct clinical phenotypes may emerge over time.

## 4. Circuit-Level and Neuroanatomical Parallels

Circuit-level observations increasingly support a lifespan-oriented interpretation in which ASD and NDDs can be viewed as temporally distinct perturbations of partially overlapping neural systems, rather than as categorically unrelated entities. In ASD, convergent evidence points to a characteristic profile of network dysconnectivity, typically described as reduced long-range integrative connectivity alongside atypical short-range or regional coupling within association cortices. The most consistently affected regions include the default mode, salience, and central executive networks, together with the anterior cingulate, orbitofrontal, lateral prefrontal, temporal, and parietal association cortices. Alterations within these distributed circuits have been associated with many of the core behavioral and cognitive manifestations of ASD, including deficits in social communication, executive function, cognitive flexibility, language, motor coordination, and sensory processing, thereby providing a functional link between network-level abnormalities and clinical phenotype [28]. Importantly, large-scale connectome analyses and meta-analytic studies indicate that ASD cannot be reduced to a simple model of global “hyper-” or “hypo-connectivity.” Instead, it exhibits spatially and developmentally heterogeneous patterns of both increased and decreased connectivity across distinct networks and stages of maturation [29,30]. This systems-level variability is consistent with postmortem and tract-level findings showing altered microstructural organization of prefrontal and cingulate pathways that support higher-order cognitive and socioemotional functions [31].

A comparable network-based perspective is well established in NDDs, where disease processes preferentially affect distributed functional systems and progressively disrupt network organization. In AD, early alterations in the default mode network (DMN) and related intrinsic connectivity architectures closely track disease risk and clinical progression, with evidence extending beyond the DMN to multiple large-scale networks [32,33]. Across the FTD spectrum, this disruption is often conceptualized as a network-level collapse, characterized by reduced segregation between functional systems and loss of integrative capacity as degeneration advances [34]. In behavioral-variant FTD, selective vulnerability of the salience network aligns closely with the syndrome’s hallmark socioemotional and behavioral changes [35].

Similarly, PD—traditionally defined by nigrostriatal dopaminergic degeneration—is increasingly understood as a systems-level disorder involving progressive alterations in large-scale connectivity that contribute to both motor and non-motor symptoms [36,37]. These observations do not imply that ASD and NDDs share identical connectivity signatures. Rather, they suggest a unifying principle: clinical phenotypes may emerge when either developmental or degenerative processes destabilize common network constraints that normally support integration, segregation, and adaptive flexibility across the lifespan.

Within this framework, certain anatomical structures emerge as key nodes of vulnerability and integration. Among these, the cerebellum occupies a central position due to its extensive closed-loop connectivity with cerebral association cortices, its early and prolonged developmental trajectory, and its role in coordinating sensorimotor, cognitive, and socioemotional functions. Disruption of cerebellar circuitry therefore has the potential to propagate broadly across distributed networks, amplifying both early neurodevelopmental perturbations and later neurodegenerative processes. Consistent with this view, the cerebellum represents one of the most reproducible anatomical substrates implicated in ASD and an increasingly recognized contributor to NDD symptomatology [3].

In ASD, postmortem studies have consistently reported abnormalities in Purkinje cells, while neuroimaging studies reveal structural and functional cerebellar alterations associated with social, cognitive, and motor features [38,39]. Experimental models further support a causal role, showing that selective disruption of Purkinje cell activity is sufficient to induce autistic-like behaviors and widespread circuit changes, highlighting the importance of cerebellar output in shaping cortical development [40]. Clinical observations reinforce this developmental sensitivity: early cerebellar injury, particularly in preterm infants, is associated with increased risk of neurodevelopmental impairments and elevated autistic traits, consistent with a time-dependent role in establishing cerebrocerebellar connectivity during critical periods [41,42]. Despite these similarities, these cerebellar abnormalities should not be interpreted as evidence of an identical pathogenic mechanism in ASD and neurodegenerative disorders. Rather, Purkinje cell alterations in ASD are thought to primarily reflect disrupted neurodevelopment and circuit formation, whereas similar findings in neurodegenerative diseases generally result from progressive neuronal degeneration occurring later in life. Thus, the overlap is more appropriately interpreted as evidence of shared biological vulnerability within cerebellar circuits than of a common pathogenic sequence or inevitable disease continuum.

In NDDs, cerebellar involvement is increasingly recognized across multiple conditions. In PD, cerebellar circuits interact closely with basal ganglia loops, contributing not only to motor symptoms such as tremor and gait disturbance but also to deficits in cognitive control and sensorimotor integration [43,44]. Similar patterns extend to atypical parkinsonian syndromes, where cerebello-cortical disconnection contributes to complex clinical presentations [45]. In AD and mild cognitive impairment, structural and functional cerebellar changes are increasingly documented and may reflect both the spread of pathology and compensatory network recruitment [46,47]. In ALS, evidence from neuroimaging and postmortem studies supports cerebellar involvement within a broader multisystem degenerative process [48]. Together, these findings position the cerebellum as a cross-lifespan integrator whose high metabolic demands, specialized plasticity, and extensive connectivity may confer heightened sensitivity to both developmental disruption and degenerative stress.

A second major point of convergence lies in the regulation of excitation–inhibition (E/I) balance and circuit homeostasis. In ASD, the E/I framework is strongly supported by both theoretical and empirical work, suggesting that increased excitation or reduced inhibitory control can destabilize neural processing [49,50]. Postmortem and molecular studies have identified alterations in GABAergic systems, including reduced expression of GAD65/67 and changes in GABA receptor–related markers in cortical and cerebellar regions [51,52], consistent with broader evidence for widespread inhibitory dysfunction [53,54]. Functionally, these alterations may contribute to increased rates of epileptiform activity, altered oscillatory dynamics, and impaired network synchronization.

In NDDs, changes in excitability are more variable, but early network hyperexcitability and interneuron dysfunction are increasingly recognized, particularly in AD, where they may contribute to cognitive impairment and facilitate the propagation of pathological processes [55,56]. Within the FTD spectrum, GABAergic deficits and altered E/I dynamics in frontal networks are associated with large-scale connectivity disruptions and behavioral syndromes [57,58]. Thus, while ASD is often framed as a condition of developmental hyperexcitability, and NDDs as involving progressive synaptic decline, both can be understood within a shared control-systems perspective: failure of inhibitory stabilization and homeostatic plasticity reduces network resilience, rendering neural systems more vulnerable to perturbations—whether these occur during development or emerge with aging.

Importantly, the strength of evidence supporting these parallels is not uniform across conditions. The ASD literature robustly supports connectivity alterations, cerebellar involvement, and E/I imbalance [30,50,59]. In contrast, direct empirical demonstrations of shared connectivity phenotypes between ASD and late-onset NDDs remain limited, and interpretations should avoid implying direct equivalence. Where mechanistic convergence is most strongly supported, it often involves pathways related to synaptic maintenance, circuit stability, and neuronal resilience—particularly in the context of PD—rather than a generalized overlap across all NDDs. Accordingly, a cautious synthesis is that ASD and NDDs exhibit convergence at the level of circuit principles—network vulnerability, hub sensitivity, cerebellar loop involvement, and E/I homeostatic regulation—while retaining distinct patterns of expression and progression. Future work will require large-scale, comparative, and longitudinal approaches to determine the extent to which these similarities reflect true shared mechanisms or parallel adaptations within different pathological contexts.

## 5. Sex and Hormonal Modulation of Shared Pathways

Sex differences represent one of the most consistent, yet mechanistically underexplored, dimensions linking ASD and NDDs. ASD shows a marked male predominance, whereas several late-life neurodegenerative conditions—most prominently AD—exhibit higher prevalence, faster progression, or distinct clinical trajectories in females. These opposing population-level patterns suggest that sex is not merely a descriptive variable, but an active biological factor that shapes vulnerability, resilience, and clinical expression across the lifespan [60,61,62]. Within this framework, sex hormones emerge as key regulators of shared cellular pathways, helping to explain how early neurodevelopmental phenotypes and late neurodegenerative processes may arise as temporally distinct outcomes of partially overlapping biological susceptibilities.

Endocrine transitions represent critical windows during which previously compensated vulnerabilities may become more evident. Puberty, for example, involves rapid changes in gonadal steroid signaling and maturation of the hypothalamic–pituitary–adrenal axis. During this period, autistic individuals show sex- and diagnosis-dependent alterations in testosterone levels and diurnal cortisol rhythms, consistent with puberty acting as a phase of symptom modulation and altered stress responsiveness [63,64]. Later in life, menopause and andropause are associated with declines in sex steroids and shifts in hormonal balance. The endocrine dyscrasia framework and reproductive-cell cycle theory propose that while balanced hormonal signaling supports neural plasticity and cellular maintenance earlier in life, dysregulation in aging may promote aberrant cell-cycle re-entry in post-mitotic neurons, contributing to amyloid accumulation, tau pathology, neuroinflammation, and cognitive decline—hallmarks of neurodegenerative disease [62]. From a lifespan perspective, the decline in estrogenic support during menopause may unmask vulnerabilities that were previously buffered, offering a plausible explanation for the increased susceptibility of females to late-life neurodegeneration despite their relative protection from ASD diagnosis during early development.

Sex steroids exert broad and pleiotropic effects on brain development, beginning prenatally and extending through early postnatal life. These hormones influence key processes such as neuronal proliferation, migration, synaptogenesis, and the establishment of excitation–inhibition balance [65]. Variations in prenatal androgen exposure have been linked to long-term, sex-specific differences in intrinsic functional connectivity, particularly within default mode network subsystems. Notably, fetal testosterone levels predict altered connectivity patterns in males but not females, suggesting a heightened sensitivity of male neurodevelopmental trajectories to androgenic signaling [66]. Complementary mechanistic insights arise from human neural stem cell models, where dihydrotestosterone has been shown to dysregulate transcriptional programs involved in neurogenesis and cortical patterning, including genes such as *MEF2C* that are also implicated in ASD-related pathways [66,67]. These findings support the idea that early endocrine environments can establish long-lasting “set points” in neural circuit organization and cellular stress responses, potentially creating latent vulnerabilities that may only become apparent under later-life challenges.

A central interface through which sex biology may influence both ASD and neurodegeneration is the neuroimmune system, particularly microglia. These cells play a critical role in synapse formation, pruning, and maintenance, and are increasingly recognized as both targets and mediators of sex steroid signaling. Notably, microglia exhibit pronounced sex differences in developmental trajectory and immune reactivity [60]. Mitochondrial function within microglia appears to be a key mechanism linking hormonal signaling to immune–synaptic interactions. Following immune activation, male microglia show a marked reduction in the expression of genes related to the mitochondrial electron transport chain, whereas female microglia maintain relatively stable expression profiles. This divergence suggests that males may be more susceptible to energy-related disruptions in microglial function during inflammatory states. Given that synaptic refinement is an energy-intensive process tightly coupled to microglial activity, a male-biased reduction in mitochondrial capacity could amplify synaptic dysregulation during critical developmental windows, thereby increasing susceptibility to ASD-related phenotypes. In contrast, females may benefit from relative early-life protection through preserved microglial mitochondrial function and broader estrogen-mediated buffering mechanisms [68].

Mitochondrial vulnerabilities extend beyond microglia to systemic bioenergetics. In human studies, certain ASD subgroups—particularly those with neurodevelopmental regression—exhibit elevated basal respiratory rates and mitochondria that are highly sensitive to physiological stress. Interestingly, similar bioenergetic profiles have been observed in first-degree relatives, suggesting heritable or shared environmental influences on mitochondrial stress sensitivity [69]. Once again, sex differences provide important context: females often demonstrate greater mitochondrial reserve capacity, consistent with the role of estrogens in enhancing mitochondrial efficiency, antioxidant defenses, and quality control mechanisms. These features are directly relevant to neurodegenerative biology, where impaired mitochondrial function and defective autophagy contribute to protein aggregation and neuronal vulnerability. Within a lifespan framework, sex hormone-mediated support of mitochondrial resilience may initially buffer ASD-associated vulnerabilities, but this protection may diminish over time, particularly during endocrine transitions such as menopause [70].

Taken together, sex and hormonal biology provide a unifying lens through which ASD and NDDs can be interpreted as dynamic expressions of a shared biological architecture [71]. During early development, androgenic influences and perinatal endocrine events may increase male susceptibility to immune–synaptic perturbations and circuit-level divergence. In contrast, estrogen-associated mechanisms may confer relative resilience in females, even when underlying biological alterations are present. With aging, the progressive decline in sex steroid signaling—especially estrogen—may reduce mitochondrial, immune, and proteostatic resilience, shifting vulnerability toward females and facilitating neurodegenerative processes. Thus, rather than conferring fixed risk, sex hormones appear to redistribute vulnerability across the lifespan, with important implications for sex-informed mechanistic models, longitudinal risk assessment, and the development of targeted preventive and therapeutic strategies.

## 6. Clinical and Epidemiological Intersections

Population-level evidence increasingly indicates that ASD is associated with a measurable burden of age-related neurological outcomes, supporting a lifespan perspective in which neurodevelopmental conditions intersect with neurodegenerative trajectories. Large-scale healthcare analyses now converge on the observation that autistic adults exhibit elevated rates of AD and related dementias (ADRD). A nationwide study of Medicaid claims encompassing more than 1.2 million adults aged 30–64 years demonstrated that individuals with ASD are significantly more likely to receive a diagnosis of early-onset dementia compared with the general population [72]. After adjustment for relevant covariates, dementia risk was approximately doubled in adults with ASD alone and nearly tripled in those with co-occurring intellectual disability, with prevalence estimates of 4.04% and 5.22%, respectively, versus 0.97% in adults without ASD or intellectual disability. These findings challenge earlier notions that ASD might confer protection against cognitive decline and instead point to an increased vulnerability to premature neurodegenerative outcomes. Complementary longitudinal analyses in publicly insured cohorts further indicate that the burden of ADRD rises as autistic populations age, reinforcing the robustness of this epidemiological signal [73].

Parallel patterns are emerging in relation to parkinsonian disorders. Although findings vary across methodologies, multiple independent lines of evidence suggest an increased burden of PD and related syndromes in autistic adults. These include higher rates of PD diagnoses in Medicare-based datasets among older autistic individuals [74], a notable prevalence of clinically defined parkinsonism in middle-aged and older autistic cohorts—including individuals without exposure to neuroleptic medications [75]—and population-based studies demonstrating an increased PD risk associated with ASD that persists after controlling for psychiatric comorbidities and medication use [76]. Importantly, symptom-oriented investigations indicate that parkinsonian features in ASD are not incidental findings. Instead, they are associated with clinically meaningful outcomes, including reduced quality of life, sleep disturbances, and increased neuropsychiatric burden—features that overlap with prodromal and non-motor phases of PD [77].

One emerging hypothesis that may help explain this epidemiological association involves the gut–brain axis. Alterations in gut microbiota composition and microbiota-derived metabolites have been implicated in both ASD and Parkinson’s disease through mechanisms involving chronic gastrointestinal dysfunction, immune activation, oxidative stress, mitochondrial dysfunction, and altered intestinal permeability. Recent work has proposed that early-life dysbiosis in ASD could increase later susceptibility to Parkinson’s disease, potentially facilitating α-synuclein pathology through gut–brain signaling pathways. Although these findings provide a biologically plausible framework linking ASD and neurodegeneration, current evidence remains largely mechanistic and epidemiological. Consequently, the gut microbiota should be considered an emerging contributor to shared biological vulnerability rather than evidence of a causal developmental trajectory [78,79].

Taken together, these clinical and epidemiological observations support a reframing of ASD as a condition that extends beyond early neurodevelopment into later-life neurological health. Elevated risks for dementia and parkinsonian disorders, along with earlier-than-expected onset and distinct patterns of cognitive and motor decline, suggest that at least a subset of autistic individuals may carry latent vulnerabilities that become more apparent with aging. Within the continuum framework advanced in this work, these findings do not imply that ASD inevitably progresses to neurodegeneration. Rather, they indicate that shared biological susceptibilities—modulated by factors such as intellectual disability, comorbid conditions, and environmental exposures—may shape long-term outcomes.

From a clinical and public health perspective, this intersection highlights important gaps in current care models. The traditional focus on pediatric and early developmental stages does not adequately address the evolving neurological needs of autistic individuals across adulthood and aging. These emerging data support the need for lifespan-oriented monitoring strategies, the establishment of normative aging trajectories specific to ASD populations, and the integration of autism into adult neurology and geriatric care frameworks. Such an approach would not only facilitate earlier detection of neurodegenerative risk but also inform targeted interventions and health policy planning as autistic cohorts increasingly transition into older adulthood.

## 7. Discussion: Autism and Neurodegeneration Across the Lifespan

The central question posed in this manuscript—whether ASD and NDDs are best understood as distinct conditions or as expressions of a shared biological continuum—emerges not as a dichotomy to be resolved, but as a framework through which diverse lines of evidence can be integrated. Across genetic, molecular, cellular, circuit-level, and epidemiological domains, the data reviewed here converge on a consistent theme: ASD and NDDs are not biologically isolated entities, but are connected through partially overlapping architectures of vulnerability that unfold across the lifespan. This convergence does not imply equivalence or inevitability. Rather, it suggests that early neurodevelopmental perturbations may, under specific conditions, bias neural systems toward increased susceptibility to later-life dysfunction, particularly in the context of cumulative oxidative stress, declining proteostatic capacity, mitochondrial inefficiency, and age-related immune dysregulation.

Adopting a lifespan-based perspective reframes ASD from a static childhood diagnosis into a dynamic neurobiological trajectory shaped by continuous interactions between genetic predisposition, environmental exposures, and cellular resilience mechanisms. Within this view, processes that disrupt early synaptic refinement, microglial maturation, calcium homeostasis, or mitochondrial buffering are not confined to development alone; they may also establish latent vulnerabilities that influence how neural systems respond to aging and physiological stress. Importantly, this relationship is not unidirectional. Insights from NDD research—particularly in areas such as proteostasis, mitochondrial quality control, and neuroimmune regulation—may inform strategies aimed at early stabilization or prevention in ASD, highlighting the value of cross-disciplinary integration.

This unified framework carries significant translational and public health implications. It supports the development of longitudinal biomarkers capable of tracking vulnerability and resilience across decades, encourages interventions that target core biological systems during critical windows of development and aging, and underscores the need to anticipate the clinical trajectories of autistic individuals as they transition into later adulthood. In this context, ASD becomes relevant not only to pediatric care but also to adult neurology and geriatric medicine, necessitating a shift toward integrated, lifespan-oriented healthcare models.

Collectively, the evidence assembled in this review supports a conceptual reorientation. ASD and NDDs, long positioned as neurobiological opposites defined by distinct temporal windows, can be more productively understood as temporally differentiated expressions of shared constraints in synaptic regulation, mitochondrial metabolism, proteostasis, and neuroimmune balance. Recognizing this continuum does not diminish the clinical distinctions between early-onset and late-onset conditions. Instead, it provides a more coherent biological framework for understanding how vulnerability is distributed, compensated, and eventually expressed over time.

Embracing this perspective opens new avenues for research and clinical practice. It encourages earlier identification of at-risk subgroups, promotes strategies aimed at enhancing long-term neural resilience, and reframes therapeutic goals to extend beyond symptom management toward sustained brain health. Ultimately, viewing neurodevelopment and neurodegeneration as interconnected processes offers a more integrative model of brain biology—one in which development, maintenance, and decline are not isolated stages, but interdependent components of a continuous trajectory shaped by shared molecular determinants.

## 8. Limitations and Future Perspectives

Although growing evidence supports the existence of shared biological mechanisms between ASD and neurodegenerative disorders, several important limitations should be acknowledged. Much of the current evidence derives from cross-sectional clinical studies, experimental animal models, or independent investigations of individual disorders rather than longitudinal studies directly examining the relationship between neurodevelopmental and neurodegenerative processes across the lifespan. Furthermore, ASD represents a highly heterogeneous condition encompassing diverse genetic, molecular, and clinical subgroups, making it unlikely that a single biological mechanism or developmental trajectory applies uniformly to all affected individuals. Consequently, the concept of a shared biological continuum should be interpreted as a framework for understanding convergent biological pathways rather than evidence of an inevitable progression from ASD to neurodegenerative disease.

Additional challenges arise from differences in study design, diagnostic criteria, patient selection, and methodological approaches across the literature, which complicate direct comparisons among studies. Although several molecular pathways—including mitochondrial dysfunction, oxidative stress, impaired autophagy, neuroinflammation, cerebellar dysfunction, and the gut–brain axis—have been independently implicated in both ASD and neurodegenerative disorders, their temporal interactions, relative contributions, and causal relationships remain incompletely understood. Establishing whether these mechanisms represent common biological vulnerabilities or disease-specific adaptations will require further investigation.

Future research should prioritize large-scale longitudinal studies that follow individuals with ASD from childhood through aging while integrating clinical, neuroimaging, genetic, epigenetic, transcriptomic, metabolomic, microbiome, and other approaches. Such studies will be essential to identify biomarkers of neuronal vulnerability, define biologically meaningful ASD subgroups, and determine whether particular molecular signatures confer increased susceptibility to neurodegenerative disorders later in life. Greater integration of experimental models with human longitudinal cohorts will also improve our understanding of the mechanisms linking early neurodevelopmental alterations with age-related neuronal dysfunction.

Ultimately, clarifying whether ASD and neurodegenerative disorders represent distinct clinical entities connected by convergent biological pathways or different manifestations of a broader spectrum of neuronal vulnerability will require multidisciplinary collaboration spanning developmental neuroscience, neurology, psychiatry, genetics, systems biology, and computational modeling. Advancing this knowledge may facilitate earlier identification of individuals at increased neurological risk and support the development of precision medicine strategies aimed at preserving brain health throughout the lifespan.

## Data Availability

No new data were created or analyzed in this study. Data sharing is not applicable to this article.

## References

[1] Valaparambil K.A., Sundaram S. (2025). Autism Spectrum Disorder and Epilepsy: Point of Convergence or Divergence. Int. J. Epilepsy.

[2] Zoghbi H.Y., Bear M.F. (2012). Synaptic Dysfunction in Neurodevelopmental Disorders Associated with Autism and Intellectual Disabilities. Cold Spring Harb. Perspect. Biol..

[3] Manzo J., Hernández-Aguilar M.E., Toledo-Cárdenas M.R., Herrera-Covarrubias D., Coria-Avila G.A., Libreros-Jiménez H.M., Fernández-Cañedo L., Ortega-Pineda L.A. (2025). The Long and Winding Road to Understanding Autism. NeuroSci.

[4] Boehm J. (2013). A ‘Danse Macabre’: Tau and Fyn in STEP with Amyloid Beta to Facilitate Induction of Synaptic Depression and Excitotoxicity. Eur. J. Neurosci..

[5] Pan L., Li C., Meng L., Tian Y., He M., Yuan X., Zhang G., Zhang Z., Xiong J., Chen G. (2022). Tau Accelerates α-Synuclein Aggregation and Spreading in Parkinson’s Disease. Brain.

[6] Ojaimi Y.A., Dangoumau A., Alarcan H., Hergesheimer R., Vourc’h P., Corcia P., Lanznaster D., Blasco H. (2022). TAR DNA-Binding Protein of 43 kDa (TDP-43) and Amyotrophic Lateral Sclerosis (ALS): A Promising Therapeutic Target. Expert Opin. Ther. Targets.

[7] Gitler A.D., Dhillon P., Shorter J. (2017). Neurodegenerative Disease: Models, Mechanisms, and a New Hope. Dis. Model. Mech..

[8] Kern J.K., Geier D.A., Sykes L.K., Geier M.R. (2013). Evidence of Neurodegeneration in Autism Spectrum Disorder. Transl. Neurodegener..

[9] Sragovich S., Merenlender-Wagner A., Gozes I. (2017). *ADNP* Plays a Key Role in Autophagy: From Autism to Schizophrenia and Alzheimer’s Disease. BioEssays.

[10] Erbescu A., Papuc S.M., Budișteanu M., Dobre M., Iliescu C., Hinescu M.E., Arghir A., Neagu M. (2025). Rare Copy Number Variants Intersecting Parkinson’s-Associated Genes in a Cohort of Children with Autism Spectrum Disorders. Neurosci. Insights.

[11] Frye R.E. (2020). Mitochondrial Dysfunction in Autism Spectrum Disorder: Unique Abnormalities and Targeted Treatments. Semin. Pediatr. Neurol..

[12] Lasser M., Sun N., Xu Y., Wang S., Drake S., Law K., Gonzalez S., Wang B., Drury V., Castillo O. (2023). Pleiotropy of Autism-Associated Chromatin Regulators. Development.

[13] Sugathan A., Biagioli M., Golzio C., Erdin S., Blumenthal I., Manavalan P., Ragavendran A., Brand H., Lucente D., Miles J. (2014). *CHD8* Regulates Neurodevelopmental Pathways Associated with Autism Spectrum Disorder in Neural Progenitors. Proc. Natl. Acad. Sci. USA.

[14] Gompers A.L., Su-Feher L., Ellegood J., Copping N.A., Riyadh M.A., Stradleigh T.W., Pride M.C., Schaffler M.D., Wade A.A., Catta-Preta R. (2017). Germline Chd8 Haploinsufficiency Alters Brain Development in Mouse. Nat. Neurosci..

[15] Ham A., Chang A.Y., Li H., Bain J.M., Goldman J.E., Sulzer D., Veenstra-VanderWeele J., Tang G. (2025). Impaired Macroautophagy Confers Substantial Risk for Intellectual Disability in Children with Autism Spectrum Disorders. Mol. Psychiatry.

[16] Sokol D.K., Maloney B., Long J.M., Ray B., Lahiri D.K. (2011). Autism, Alzheimer Disease, and Fragile X. Neurology.

[17] Bleuzé L., Triaca V., Borreca A. (2021). FMRP-Driven Neuropathology in Autistic Spectrum Disorder and Alzheimer’s Disease: A Losing Game. Front. Mol. Biosci..

[18] Nadeem M.S., Hosawi S., Alshehri S., Ghoneim M.M., Imam S.S., Murtaza B.N., Kazmi I. (2021). Symptomatic, Genetic, and Mechanistic Overlaps between Autism and Alzheimer’s Disease. Biomolecules.

[19] Garcia-Junco-Clemente P., Golshani P. (2014). *PTEN*: A Master Regulator of Neuronal Structure, Function, and Plasticity. Commun. Integr. Biol..

[20] Gilbert J., Man H.-Y. (2017). Fundamental Elements in Autism: From Neurogenesis and Neurite Growth to Synaptic Plasticity. Front. Cell. Neurosci..

[21] Banerjee R., Beal M.F., Thomas B. (2010). Autophagy in Neurodegenerative Disorders: Pathogenic Roles and Therapeutic Implications. Trends Neurosci..

[22] Rossignol D.A., Frye R.E. (2012). Mitochondrial Dysfunction in Autism Spectrum Disorders: A Systematic Review and Meta-Analysis. Mol. Psychiatry.

[23] Swerdlow N.S., Wilkins H.M. (2020). Mitophagy and the Brain. Int. J. Mol. Sci..

[24] Youle R.J., Narendra D.P. (2011). Mechanisms of Mitophagy. Nat. Rev. Mol. Cell Biol..

[25] Yang Z., Luo Y., Yang Z., Liu Z., Li M., Wu X., Chen L., Xin W. (2025). Mitochondrial Dynamics Dysfunction and Neurodevelopmental Disorders: From Pathological Mechanisms to Clinical Translation. Neural Regen. Res..

[26] Kovacheva E., Gevezova M., Mehterov N., Kazakova M., Sarafian V. (2025). The Intersection of Mitophagy and Autism Spectrum Disorder: A Systematic Review. Int. J. Mol. Sci..

[27] Eissa N., Sadeq A., Sasse A., Sadek B. (2020). Role of Neuroinflammation in Autism Spectrum Disorder and the Emergence of Brain Histaminergic System. Lessons Also for BPSD?. Front. Pharmacol..

[28] Müller R.-A., Fishman I. (2018). Brain Connectivity and Neuroimaging of Social Networks in Autism. Trends Cogn. Sci..

[29] Ilioska I., Oldehinkel M., Llera A., Chopra S., Looden T., Chauvin R., Rooij D.V., Floris D.L., Tillmann J., Moessnang C. (2023). Connectome-Wide Mega-Analysis Reveals Robust Patterns of Atypical Functional Connectivity in Autism. Biol. Psychiatry.

[30] Hull J.V., Dokovna L.B., Jacokes Z.J., Torgerson C.M., Irimia A., Horn J.D.V. (2017). Resting-State Functional Connectivity in Autism Spectrum Disorders: A Review. Front. Psychiatry.

[31] Zikopoulos B., Barbas H. (2013). Altered Neural Connectivity in Excitatory and Inhibitory Cortical Circuits in Autism. Front. Hum. Neurosci..

[32] Pievani M., Haan W., de Wu T., Seeley W.W., Frisoni G.B. (2011). Functional Network Disruption in the Degenerative Dementias. Lancet Neurol..

[33] Badhwar A., Tam A., Dansereau C., Orban P., Hoffstaedter F., Bellec P. (2017). Resting-State Network Dysfunction in Alzheimer’s Disease: A Systematic Review and Meta-Analysis. Alzheimer’s Dement. Diagn. Assess. Dis. Monit..

[34] Brown J.A., Lee A.J., Fernhoff K., Pistone T., Pasquini L., Wise A.B., Staffaroni A.M., Mandelli M.L., Lee S.E., Boxer A.L. (2025). Functional Network Collapse in Neurodegenerative Disease. Nat. Commun..

[35] Ferreira L.K., Lindberg O., Santillo A.F., Wahlund L. (2022). Functional Connectivity in Behavioral Variant Frontotemporal Dementia. Brain Behav..

[36] Stoessl A.J., Lehericy S., Strafella A.P. (2014). Imaging Insights into Basal Ganglia Function, Parkinson’s Disease, and Dystonia. Lancet.

[37] Churchill L., Ignatavicius A., Konuri A., Anderson J., Taylor N., Lewis S.J.G., Matar E. (2025). Functional Magnetic Resonance Imaging (fMRI) Signatures of Progression and Phenoconversion in Prodromal Synucleinopathies. Mov. Disord..

[38] Biswas M.S., Roy S.K., Hasan R., Pk M.M.U. (2024). The Crucial Role of the Cerebellum in Autism Spectrum Disorder: Neuroimaging, Neurobiological, and Anatomical Insights. Health Sci. Rep..

[39] Brandenburg C., Griswold A.J., Booven D.J.V., Kilander M.B.C., Frei J.A., Nestor M.W., Dykxhoorn D.M., Pericak-Vance M.A., Blatt G.J. (2022). Transcriptomic Analysis of Isolated and Pooled Human Postmortem Cerebellar Purkinje Cells in Autism Spectrum Disorders. Front. Genet..

[40] Tsai P.T., Hull C., Chu Y., Greene-Colozzi E., Sadowski A.R., Leech J.M., Steinberg J., Crawley J.N., Regehr W.G., Sahin M. (2012). Autistic-like Behavior and Cerebellar Dysfunction in Purkinje Cell *Tsc1* Mutant Mice. Nature.

[41] Limperopoulos C., Bassan H., Gauvreau K., Robertson R.L., Sullivan N.R., Benson C.B., Avery L., Stewart J., Soul J.S., Ringer S.A. (2007). Does Cerebellar Injury in Premature Infants Contribute to the High Prevalence of Long-Term Cognitive, Learning, and Behavioral Disability in Survivors?. Pediatrics.

[42] Stoodley C.J., Limperopoulos C. (2016). Structure–Function Relationships in the Developing Cerebellum: Evidence from Early-Life Cerebellar Injury and Neurodevelopmental Disorders. Semin. Fetal Neonatal Med..

[43] Mirdamadi J.L. (2016). Cerebellar Role in Parkinson’s Disease. J. Neurophysiol..

[44] Pietracupa S., Ojha A., Belvisi D., Piervincenzi C., Tommasin S., Petsas N., Bartolo M.I.D., Costanzo M., Fabbrini A., Conte A. (2024). Understanding the Role of Cerebellum in Early Parkinson’s Disease: A Structural and Functional MRI Study. npj Park. Dis..

[45] Yao Q., Zhu D., Li F., Xiao C., Lin X., Huang Q., Shi J. (2017). Altered Functional and Causal Connectivity of Cerebello-Cortical Circuits between Multiple System Atrophy (Parkinsonian Type) and Parkinson’s Disease. Front. Aging Neurosci..

[46] Tang F., Zhu D., Ma W., Yao Q., Li Q., Shi J. (2021). Differences Changes in Cerebellar Functional Connectivity Between Mild Cognitive Impairment and Alzheimer’s Disease: A Seed-Based Approach. Front. Neurol..

[47] Jacobs H.I.L., Hopkins D.A., Mayrhofer H.C., Bruner E., Leeuwen F.W., van Raaijmakers W., Schmahmann J.D. (2018). The Cerebellum in Alzheimer’s Disease: Evaluating Its Role in Cognitive Decline. Brain.

[48] Chipika R.H., Mulkerrin G., Pradat P.-F., Murad A., Ango F., Raoul C., Bede P. (2022). Cerebellar Pathology in Motor Neuron Disease: Neuroplasticity and Neurodegeneration. Neural Regen. Res..

[49] Sohal V.S., Rubenstein J.L.R. (2019). Excitation-Inhibition Balance as a Framework for Investigating Mechanisms in Neuropsychiatric Disorders. Mol. Psychiatry.

[50] Rubenstein J.L.R., Merzenich M.M. (2003). Model of Autism: Increased Ratio of Excitation/Inhibition in Key Neural Systems. Genes Brain Behav..

[51] Blatt G.J., Fatemi S.H. (2011). Alterations in GABAergic Biomarkers in the Autism Brain: Research Findings and Clinical Implications. Anat. Rec..

[52] Fatemi S.H., Halt A.R., Stary J.M., Kanodia R., Schulz S.C., Realmuto G.R. (2002). Glutamic Acid Decarboxylase 65 and 67 kDa Proteins Are Reduced in Autistic Parietal and Cerebellar Cortices. Biol. Psychiatry.

[53] Wang P., Sun J. (2025). A Comprehensive Review of GABA in Autism Spectrum Disorders: Associations, Mechanisms, and Therapeutic Implications. Front. Psychiatry.

[54] Zhao H., Mao X., Zhu C., Zou X., Peng F., Yang W., Li B., Li G., Ge T., Cui R. (2022). GABAergic System Dysfunction in Autism Spectrum Disorders. Front. Cell Dev. Biol..

[55] Anastacio H.T.D., Matosin N., Ooi L. (2022). Neuronal Hyperexcitability in Alzheimer’s Disease: What Are the Drivers behind This Aberrant Phenotype?. Transl. Psychiatry.

[56] Kazim S.F., Seo J.H., Bianchi R., Larson C.S., Sharma A., Wong R.K.S., Gorbachev K.Y., Pereira A.C. (2021). Neuronal Network Excitability in Alzheimer’s Disease: The Puzzle of Similar versus Divergent Roles of Amyloid β and Tau. eNeuro.

[57] Murley A.G., Rouse M.A., Jones P.S., Ye R., Hezemans F.H., O’Callaghan C., Frangou P., Kourtzi Z., Rua C., Carpenter T.A. (2020). GABA and Glutamate Deficits from Frontotemporal Lobar Degeneration Are Associated with Disinhibition. Brain.

[58] Adams N.E., Hughes L.E., Rouse M.A., Phillips H.N., Shaw A.D., Murley A.G., Cope T.E., Bevan-Jones W.R., Passamonti L., Street D. (2021). GABAergic Cortical Network Physiology in Frontotemporal Lobar Degeneration. Brain.

[59] D’Mello A.M., Stoodley C.J. (2015). Cerebro-Cerebellar Circuits in Autism Spectrum Disorder. Front Neurosci..

[60] McCarthy M.M., Wright C.L. (2017). Convergence of Sex Differences and the Neuroimmune System in Autism Spectrum Disorder. Biol. Psychiatry.

[61] Kolahchi Z., Henkel N., Eladawi M.A., Villarreal E.C., Kandimalla P., Lundh A., McCullumsmith R.E., Cuevas E. (2024). Sex and Gender Differences in Alzheimer’s Disease: Genetic, Hormonal, and Inflammation Impacts. Int. J. Mol. Sci..

[62] Atwood C.S., Bowen R.L. (2015). The Endocrine Dyscrasia That Accompanies Menopause and Andropause Induces Aberrant Cell Cycle Signaling That Triggers Re-Entry of Post-Mitotic Neurons into the Cell Cycle, Neurodysfunction, Neurodegeneration and Cognitive Disease. Horm. Behav..

[63] Muscatello R.A., Rafatjoo E., Mirpuri K.K., Kim A., Vandekar S., Corbett B.A. (2022). Salivary Testosterone in Male and Female Youth with and without Autism Spectrum Disorder: Considerations of Development, Sex, and Diagnosis. Mol. Autism.

[64] Corbett B.A., McGonigle T., Muscatello R.A., Liu J., Vandekar S. (2023). The Developmental Trajectory of Diurnal Cortisol in Autistic and Neurotypical Youth. Dev. Psychopathol..

[65] Victor X.D.-E., Barradas-Moctezuma M., Herrera-Covarrubias D., Manzo J., Genaro A.C.-A. (2020). Nature and Nurture of Sexual Partner Preference: Teachings from Prenatal Administration of Acetaminophen in Male Rats. Horm. Behav..

[66] Lombardo M.V., Auyeung B., Pramparo T., Quartier A., Courraud J., Holt R.J., Waldman J., Ruigrok A.N.V., Mooney N., Bethlehem R.A.I. (2020). Sex-Specific Impact of Prenatal Androgens on Social Brain Default Mode Subsystems. Mol. Psychiatry.

[67] Hu V.W., Sarachana T., Sherrard R.M., Kocher K.M. (2015). Investigation of Sex Differences in the Expression of *RORA* and Its Transcriptional Targets in the Brain as a Potential Contributor to the Sex Bias in Autism. Mol. Autism.

[68] Bordt E.A., Moya H.A., Jo Y.C., Ravichandran C.T., Bankowski I.M., Ceasrine A.M., McDougle C.J., Carlezon W.A., Bilbo S.D. (2024). Gonadal Hormones Impart Male-Biased Behavioral Vulnerabilities to Immune Activation via Microglial Mitochondrial Function. Brain Behav. Immun..

[69] Frye R.E., McCarty P.J., Werner B.A., Rose S., Scheck A.C. (2024). Bioenergetic Signatures of Neurodevelopmental Regression. Front. Physiol..

[70] Mohajeri M., Martín-Jiménez C., Barreto G.E., Sahebkar A. (2019). Effects of Estrogens and Androgens on Mitochondria under Normal and Pathological Conditions. Prog. Neurobiol..

[71] Shapira G., Karmon G., Hacohen-Kleiman G., Ganaiem M., Shazman S., Theotokis P., Grigoriadis N., Shomron N., Gozes I. (2025). *ADNP* Is Essential for Sex-Dependent Hippocampal Neurogenesis, through Male Unfolded Protein Response and Female Mitochondrial Gene Regulation. Mol. Psychiatry.

[72] Vivanti G., Tao S., Lyall K., Robins D.L., Shea L.L. (2021). The Prevalence and Incidence of Early-onset Dementia among Adults with Autism Spectrum Disorder. Autism Res..

[73] Tewolde S., Rosenberg S.B., Estrada J.A.G., Jimenez M.P., Scott A., Higgins A., Rubenstein E. (2025). Epidemiology of Alzheimer Disease and Related Dementia Among Medicare and Medicaid Enrolled Autistic Adults, 2011–2019. Autism Res..

[74] Hand B.N., Angell A.M., Harris L., Carpenter L.A. (2020). Prevalence of Physical and Mental Health Conditions in Medicare-Enrolled, Autistic Older Adults. Autism.

[75] Starkstein S., Gellar S., Parlier M., Payne L., Piven J. (2015). High Rates of Parkinsonism in Adults with Autism. J. Neurodev. Disord..

[76] Yin W., Reichenberg A., Beeri M.S., Levine S.Z., Ludvigsson J.F., Figee M., Sandin S. (2025). Risk of Parkinson Disease in Individuals With Autism Spectrum Disorder. JAMA Neurol..

[77] Wallace G.L., Said A.J., McQuaid G.A. (2025). Elevated Parkinsonism Symptoms in Autism during Middle and Older Adulthood Are Linked with Psychosocial, Physical Health, and Mental Health Outcomes. Autism Res..

[78] Bicknell B., Liebert A., Borody T., Herkes G., McLachlan C., Kiat H. (2023). Neurodegenerative and Neurodevelopmental Diseases and the Gut-Brain Axis: The Potential of Therapeutic Targeting of the Microbiome. Int. J. Mol. Sci..

[79] Zhao M.-M., Hashimoto K., Yang J.-J. (2026). Early-Life Microbiota Dysbiosis as a Link Between Autism Spectrum Disorder and Parkinson’s Disease. Mol. Psychiatry.

